# Effects of Fabrication Methods on the Performance of Luminescent Solar Concentrators Based on Doped Polymer Optical Fibers

**DOI:** 10.3390/polym13030424

**Published:** 2021-01-28

**Authors:** Eneko Arrospide, María Asunción Illarramendi, Igor Ayesta, Nekane Guarrotxena, Olga García, Joseba Zubia, Gaizka Durana

**Affiliations:** 1 Department of Applied Mathematics, Engineering School of Bilbao, University of the Basque Country (UPV/EHU), Plaza Ingeniero Torres Quevedo 1, 48013 Bilbao, Spain; igor.ayesta@ehu.eus; 2 Department of Applied Physics I, Engineering School of Bilbao, University of the Basque Country (UPV/EHU), Plaza Ingeniero Torres Quevedo 1, 48013 Bilbao, Spain; ma.illarramendi@ehu.eus; 3 Instituto de Ciencia y Tecnología de Polímeros, Consejo Superior de Investigaciones Científicas (ICTP-CSIC), Juan de la Cierva 3, 28006 Madrid, Spain; nekane@ictp.csic.es (N.G.); ogarcia@ictp.csic.es (O.G.); 4 Department of Communications Engineering, Engineering School of Bilbao, University of the Basque Country (UPV/EHU), Plaza Ingeniero Torres Quevedo 1, 48013 Bilbao, Spain; joseba.zubia@ehu.eus (J.Z.); gaizka.durana@ehu.eus (G.D.)

**Keywords:** polymer optical fibers, fiber fabrication, luminescent materials, luminescent solar concentrators, green energy

## Abstract

In this work, we detail two types of fabrication processes of four polymer optical fibers doped with lumogen dyes. The fiber preforms have been manufactured with two different methods: extrusion and casting. We have compared the performance of the two types of fibers as luminescent solar concentrators by calculating their optical efficiencies and concentration factors. The obtained results show better performance for those fibers manufactured by the casting process. We have also studied the photostability of the two types of fibers doped with the dye lumogen red under solar light radiation. A high thermal stability of the doped fibers has been observed.

## 1. Introduction

Optical devices based on organic polymeric materials, such as optical communications or optoelectronics, are currently effective low-cost solutions in the photonic field [1]. In recent years, there has been a growing interest in embedding functional materials into organic solid-state matrices [2], and specifically, into polymer optical fibers (POFs) due to the multiple advantages offered by the cylindrical geometry [2]. The mechanical resistance and flexibility of POFs are greater than those of their glass counterparts, and their manufacturing technology is simpler and cheaper [3,4]. On the other hand, the much lower manufacturing temperatures of POFs make it possible to incorporate a variety of functional dopants into the fiber core, from organic dyes, ions of rare earths and quantum dots to noble metal nanoparticles. By choosing the appropriate dopant, a large variety of photonic components based on POFs can be manufactured: lasers, amplifiers, illuminators, switches, sensors or solar concentrators.

Doped POFs can be operated as luminescent solar concentrators (LSCs) because they are able to absorb sunlight through their surface, to emit at a greater wavelength and to guide the generated emission, by total internal reflection, to the fiber ends where the photovoltaic cells (PVs) are placed. The idea of concentrating sunlight using luminescent dopants embedded into transparent matrices with the objective to reduce the cost of PVs was first published in 1970 by Weber and Lambe [5]. LSCs present interesting properties, such as a sunlight capture at different angles (they do not need a solar tracking system), an invariable performance under different lighting conditions and good heat dissipation. Unlike traditional planar rectangular LSCs, cylindrical LSCs, as doped POFs, have a better coupling between their ends to the PVs and they can easily be connected to other transparent POFs to allow spatial separation between the light collection and the detection systems, if necessary. Theoretical studies have revealed that the optical concentration of a cylindrical concentrator is between 1 to 1.9 times greater than that of an equivalent flat concentrator when the emission occurs near the surface [6]. Other studies have also concluded that luminescent molecules should absorb and reemit light very close to the surface of cylindrical LSCs for greater efficiency [7,8]. On the other hand, a comparison between the performance of flat and cylindrically bent LCSs reveals that bent systems have higher electrical and optical conversion efficiency and are less sensitive to the angular distribution of the incident irradiance [9]. Solar optical efficiencies of POFs doped with europium complexes and with quantum dots present similar values to those obtained in flat concentrators [7,10]. With the aim of improving the performance of cylindrical LSCs, recent studies show that the overall optical-to-electrical conversion efficiency of multiple doped fibers with various designs rivals that of reported planar slab LSCs [11]. In particular, it has been demonstrated that the efficiency of LSCs is improved by using an array of cylindrical optical fibers [12]. More recent options are LSCs based on melt-spun POFs, which can constitute a cost-effective solution to enhance the illumination-angle dependence of solar cell power production [13]. Electrospun nanofibers incorporating nanoparticles to enhance the dye emissions can also be employed in conjunction with an organic PV [14,15]. All these properties, combined with commonly available materials and ease of processing, make doped POFs an actual attractive option in the area of LSCs [15,16,17]. Nowadays one of the main applications of LSCs is focused on the building integration of PV energy (BIPV) in modern cities, where the cost of land for the installation of ground PVs is prohibitively high and the rooftop space is too scarce to accommodate PV modules [18,19,20]. As a matter of fact, the development of light conversion systems for BIPV has encouraged the publication of a large number of research works about LSCs in the last few years [17]. On the other hand, LSCs can be used as powerful and highly adaptive photonic platforms with many potential applications other than those for BIPV, such as displays, chemical reactors, dark-field imaging or several sensing applications [15]. In this sense, fiber LSCs could allow for greater light concentration than standard planar plates, and they could contribute in the BIPV area to the transition to fully energetically sustainable architectures and in other alternative application areas.

There are different methods of fabricating POFs. The knowledge of the advantages and drawbacks of the methods is especially important, since the fabrication method affects the properties of POFs. In this work, we study the effect of the production method on the performance of doped POFs as LSCs. In particular, we report two types of fabrication processes of four polymer optical fibers doped with lumogen dyes (violet, yellow orange and red). The fiber preforms have been manufactured using two different methods: extrusion and casting. The parameters of the LSCs obtained with these two methods, such as the optical efficiency and the concentration factor, have been calculated and compared to each other. We have also studied the properties of lumogen red doped POFs manufactured by the casting process for different dye concentrations. Finally, we analyze the photostability of several POFs doped with the dye lumogen red.

## 2. Experimental

### 2.1. Fabrication of POFs 

**Figure 1:**
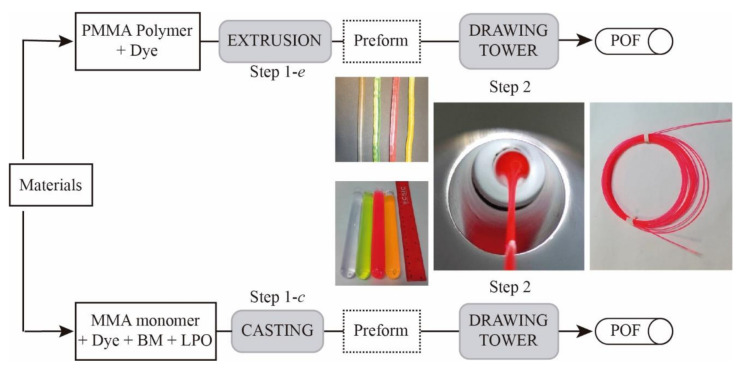
Schematic representation of the steps followed in each of the two processes for POF fabrication. Photographs of the extrusion (top) and casting (bottom) preforms, of a LR casting preform stretching in the drawing-tower and of the final LR casting fiber.

The fibers employed in this study were self-fabricated following a two-step process consisting of fabricating a preform (step 1) and then drawing it down to fiber (step 2). We used Poly(methyl-methacrylate) (PMMA) as host material and four different active dye derivatives from perylene and naphtalimide as active dopants, namely, Lumogen F Violet 570 (LV), Lumogen F Yellow 083 (LY), Lumogen F Orange 240 (LO), and Lumogen F Red 305 (LR). These Lumogen F dyes were developed by BASF (Ludwigshafen, Germany) to have high quantum yields and good photostability for use in LSCs [21]. The flow chart of the two fabrication processes is shown in Figure 1.

The preforms (step 1) were produced following two different processes: extrusion and casting. In the extrusion method (step 1-e), the doped material (the mixture of PMMA and dye) was fed into the reaction chamber of a homemade extruder, melted at 165 °C and, then, directed to the screw extruder. The material was pushed through a small nozzle, and, finally, the preform was fabricated with a diameter of 11 mm. The dye concentration was 0.003 wt% in all cases. The final material was kept at 4 °C in a refrigerator immediately prior to use. 

In the casting process (step 1-c), dopants in different concentrations (LV, LY, LO at 0.003 wt% and LR at 0.003, 0.03 and 0.01 wt%, respectively), lauroylperoxide (LPO, 0.03 mol%) and 1-butyl-mercaptan (BM, 0.2 mol%) were solved in a nitrogen saturated methyl-methacrylate (MMA) monomer solution at room temperature. After filtering the monomer solution through 0.45 μm PTFE syringe filter into borosilicate tubes of 10 mm diameter, the tubes were sealed with silicon plugs and then transferred into a programmable heating oven. The preforms were heated slowly (0.6 °C/h) to 100 °C. This temperature was kept constant for 2 days before cooling them down (5 °C/h) to 20 °C. Afterward, the final product was stored at −20 °C until further use.

Finally, in both types of fabrication, the preforms were annealed over 14 days in a climate temperature system before drawing them to only-core fibers using a drawing-tower at a maximum furnace-temperature of 185 °C [22]. The maximum diameter deviation was kept below 7%. Table 1 summarizes the main characteristics of the ten fiber samples analyzed in this work.

### 2.2. Characterization Methods

**Figure 2:**
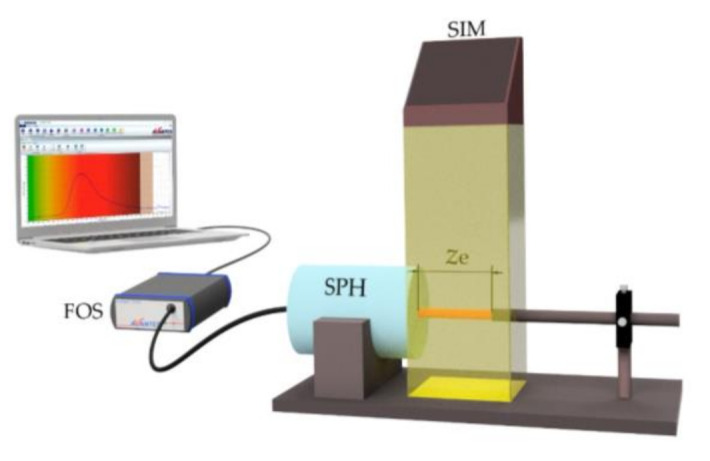
Illustration of the experimental set-up employed for the characterization of cylindrical LSCs using a solar-simulator. Legend: SIM: Solar-simulator; SPH: Integrating sphere; FOS: fiber-optic spectrometer. *z*_e_ represents the illuminated fiber length. The non-excited fiber length up to the detector *z*_ne_ is zero.

Figure 2 shows the illustration of the experimental set-up used for the characterization of cylindrical LSCs. The doped fibers were illuminated with a solar-simulator (Abet Technologies, Sunlite Model 11002, Milford, CT, USA) under the standard 1 Sun AM 1.5G solar radiation (1000 W/m^2^) which was always calibrated prior to use with a calibrated Si reference cell (Abet Technologies, Reference Cell 15150). The fibers were placed perpendicularly to the calibrated light source, allowing uniform side excitation on a total area of 5 × 5 cm^2^. The illuminated fiber length (*z*_e_) was adjusted by means of a sliding blade that was placed in the path between the solar-simulator and the doped fiber. For measuring the output intensity at one of the fibers ends, we used a calibrated fiber-spectrometer in the 350–1000 nm range (AvaSpec-ULS2048-USB2 FC/PC, Apeldoorn, The Netherlands) together with an integrating sphere with active diameter of 50 mm (Avasphere-50-IRRAD). A type-K thermocouple (0.5 °C resolution) placed very near to the fiber surfaces was used to estimate the temperature of the fibers under the solar-simulator.

The characterization of the fibers as solar concentrators was carried out by means of the optical efficiency *η*_opt_ and the concentration factor *C*. These magnitudes quantify the optical processes that take place in LSCs, as sunlight absorption, absorbed light conversion, emitted light propagation and concentration. The efficiency is the indicator that better describes the fundamental property of an LSC, namely light collection and concentration. It is defined as the ratio between the radiative power emitted from the edge of the LSC to the radiative power incident on the top surface:

(1) $$\eta_{opt}=\frac{P_{out}^{rad}}{P_{in}}.$$

The output radiative power ($P_{out}^{rad}$) is calculated by integrating the spectral curve of absolute irradiance given by the spectrometer in the spectral range corresponding to the emission band of the fiber. The radiative incident power *P*_in_ is calculated as *I*_solar_ × 2 *r* z_e_, where *I*_solar_ is the intensity of the solar-simulator (1000 W/m^2^) and 2 *r* z_e_ is the illuminated fiber surface perpendicular to the vertical direction of the incident radiation. The optical efficiency does not give any details about the large difference between the area LSC front surface exposed to solar light and the area of its edges where the concentrated emitted light is detected. For that reason, the concentration factor *C* is introduced as *C* = *η*_opt_ × *G* where *G* is the geometric gain, namely, the ratio of the input surface area to the area of edges. The concentration factor *C* can be thought of as an effective enlargement factor of an area of a PV device when it is coupled to an LSC. The concentration factor of a doped POF increases linearly with its length up to reach the saturation fiber length [23,24].

The absorption bands of the doped fibers were recorded on a Cary 50 UV–Vis spectrophotometer (Agilent Technologies, Santa Clara, CA, USA) equipped with a fiber optic coupler accessory. The optical loss coefficients of the fibers were calculated by using the side-illumination fluorescence (SIF) measurement. This non-destructive method based on transverse excitation consists in measuring the decrease of the intensity of the fluorescence spectra as the light propagation distance increases through the fibers [4]. A schematic representation of the experimental set-up used for these measurements is shown in reference [25].

## 3. Results and Discussion

### 3.1. Absorption and Emission Spectra

**Figure 3:**
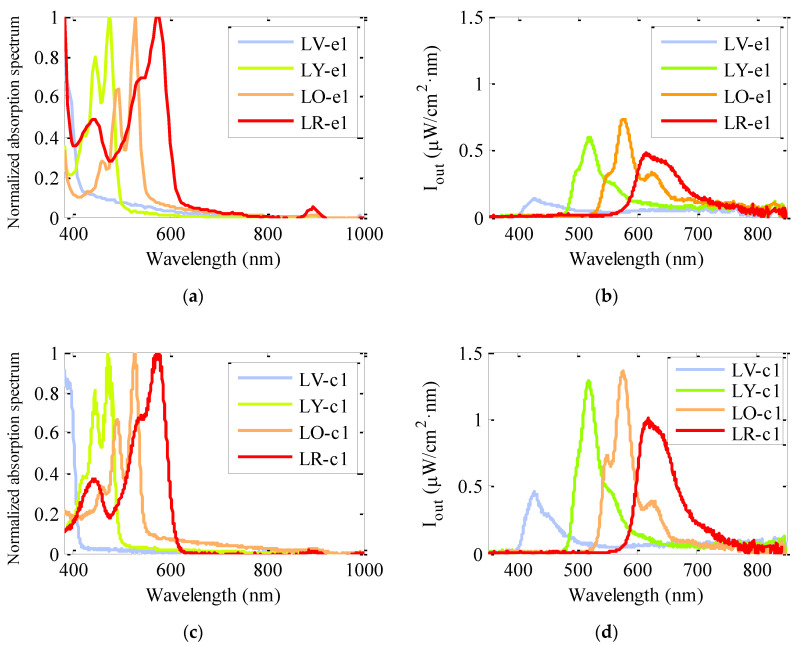
Relative absorption and absolute emission spectra of dye-doped fiber samples made by extrusion method (**a**,**b**) and casting method (**c**,**d**) (with z_e_ = 5 cm and z_ne_ = 0). The main absorption peaks for the LV, LY, LO and LR fibers are located at 392, 472, 524 and 575 nm, respectively, and the emission peaks lie at 427, 518, 575 and 616 nm.

The absorption and emission spectra of the eight fiber samples with the same dye concentration are shown in Figure 3. Four of them were made by means of the extrusion method and the other four by the casting method. The absorption spectra were measured by employing fiber samples of around 1 cm in length, so that the bands of the dopants embedded in the fiber could be detected. As can be seen, the two types of lumogen-doped fibers present very similar broad absorption bands in a wide wavelength range that is suitable to collect the solar irradiance. The absolute emission spectra, measured in μW/cm^2^·nm, were recorded by illuminating 5-cm-long fibers with the calibrated solar-simulator. The spectral shapes of the emission bands of the two types of doped fibers are similar, with the emission peaks placed at the same wavelengths for the two fabrication methods, namely 427, 518, 575 and 616 nm for the LV, LY, LO and LR fibers, respectively. These peaks are red shifted around 3 nm as compared to those of the emission spectra obtained by exciting the extrusion fibers with a laser at the maximum absorption wavelength, and with z_e_ = 1.2 mm and z_ne_ = 3.3 cm [23]. This red shift can be due to the longer propagation distances of light in the fibers in this case. Regarding the intensity values, it can be seen that the emission intensity of the four fibers made by the casting method is noticeably higher. It should be noticed that the main drawback of using organic dyes lies in the strong overlap between the absorption and emission spectra. This overlap leads to reabsorption and reemission of radiation, together with a decrease of luminescence intensity as the light propagation distance increases and some heat is generated. The overlaps corresponding to the four lumogen dyes in the two types of fibers of this work are very similar, of around 50 nm.

### 3.2. Performance under Calibrated Solar-Simulator

**Figure 4:**
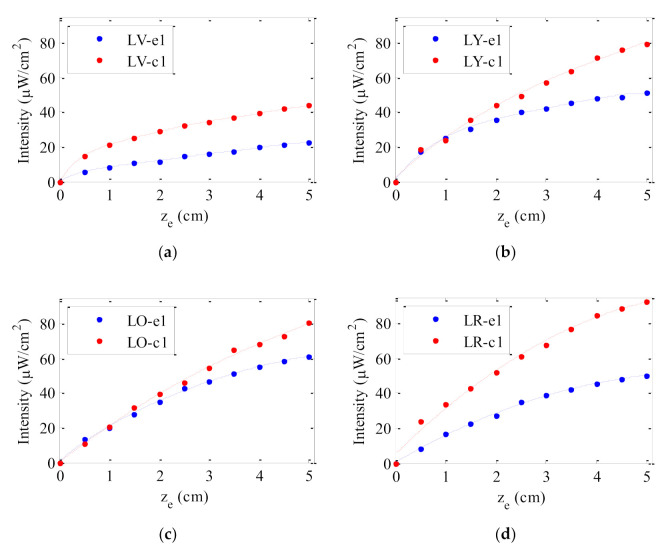
Output intensity as a function of the illuminated length on the fiber (z_e_) for (**a**) LV, (**b**) LY, (**c**) LO and (**d**) LR samples under sun-simulator lamp exposure; the solid-lines are guides to the eye.

**Figure 5:**
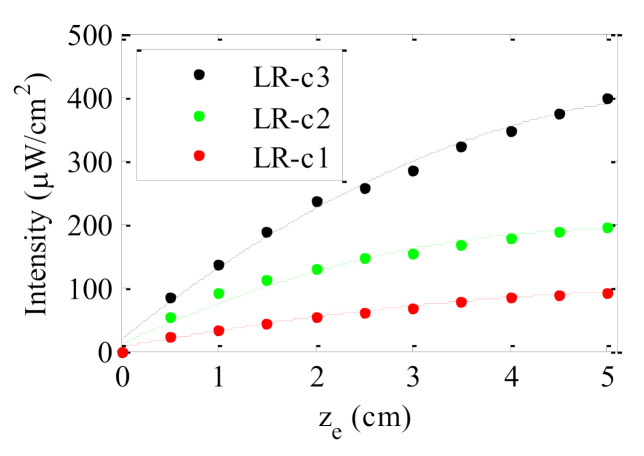
Output intensity as a function of the illuminated length (z_e_) on the fiber for the three samples doped with lumogen red (LR) under sun-simulator lamp exposure; the solid-lines are guides to the eye.

Figure 4 shows the evolution of the output intensity emitted by all the doped fibers with dye concentration 0.003 wt% as a function of the illuminated fiber length *z*_e_. In all cases, the intensity emitted was measured at one of the fiber ends without a non-excited fiber length (see Figure 2). As can be observed, for short illuminated lengths, the output signal in all samples increases, but, afterwards, it tends to saturate. The same behavior has already been detected in previous works [23,24,26]. In agreement with the results obtained in Section 3.1, the fibers made by the casting method emit higher output intensity for any illuminated fiber length. The concentration of the dopant also plays an important role in the output intensity as can be seen in Figure 5. This figure shows the results obtained for the fiber samples doped with three different concentrations of LR. As can be observed, the output intensity measured at any value of *z*_e_ increases with the dopant concentration. The results indicate that the saturation length of the fiber, namely, the length from which the output power does not increase if *z*_e_ is further increased, has not been reached for any of the concentrations employed. In such a case, an increase in the concentration is beneficial, because more sunlight can be absorbed by the fiber. However, if the concentration is so high that the fiber length exceeds the saturation length for that concentration, any increment in the concentration would spoil the performance of the fiber. These results also indicate that the three concentrations of LR analyzed in this paper are not big enough to detect a photoluminescence quenching effect due to the creation of aggregates between molecules [25]. Therefore, the solar concentrator performance of the LR-doped fibers could be improved even further by increasing the dye concentration up to reach the concentration threshold where the fluorescence quenching starts to occur. 

The output intensities and powers emitted by all the fiber samples and the corresponding optical efficiencies are displayed in Table 2. The data were obtained by illuminating a fiber length of 5 cm. The concentration factor *C* of all samples, which has been calculated from the geometric gain *G*, has also been included in the table. The values of *G* of the studied fibers are ranged between 64 and 86. On one hand, focusing in the performance of the different dyes, LR-doped fibers present the highest values of *I*_out_, *P*_out_, *η*_opt_ and *C*, and among them, the fiber made by the casting method with the highest concentration is the one that offers the highest values. On the contrary, the lowest values correspond to the fibers doped with LV. These results are mainly caused by the absorption spectra of the fibers doped with LV which lie in the near-ultraviolet spectral region where the solar radiation is weaker. The absorption spectra of the LR fibers match better with the solar radiation spectrum. This result related to the behavior of different lumogen dyes was previously detected in extrusion fibers by using a halogen lamp [23]. As we indicated in Section 3.1, the performance of luminescent solar concentrators also depends on the overlap between the absorption and emission spectra of the dye dopants. However, this fact cannot significantly affect the different behavior between the dyes since their spectral overlaps are similar.

On the other hand, comparing the performance of the two different types of fibers doped with the same dye, we notice that all the magnitudes of Table 2 corresponding to the casting fibers are approximately 1.6 times higher than those corresponding to the extrusion fibers. Since both types of fibers have the same dye at the same concentration, the better operation of the casting fibers cannot be attributed to the photoluminiscent quantum yield of the dye or to the fraction of solar light absorbed by the dye. It cannot be explained from reabsorption and reemission processes either. This better operation could be explained by taking into account the multiple energy-loss mechanisms that may occur all along the fiber lengths irrespective of the dye properties, such as (i) the losses caused by rays with angles smaller than the critical one, which will leak out when reaching the fiber surface, (ii) the light-scattering caused by impurities of the host material, and (iii) the reflection losses determined by the smoothness of the fiber surface. The energy lost by leaking (i) can be calculated from the waveguiding efficiency of the fiber, which is the portion of the emitted light that is trapped inside the waveguide. This fraction can be determined from the critical angle using the refractive index of the fiber [27]. Due to the low concentration of the dye in the PMMA fibers, the refractive index of all fibers is practically the same (n = 1.49) and, therefore, the energy loss due to this leaking effect is the same in both types of fibers. A calculation of the trapping efficiency in the analyzed fibers by taking isotropic emitters placed uniformly along the fiber yields a value of 29%, regardless of the fiber length. The remaining losses (ii and iii), related to the guiding of the light through the fibers, and to the partial reflections on the fiber surface, can be evaluated from the attenuation coefficients of the fibers.

**Figure 6:**
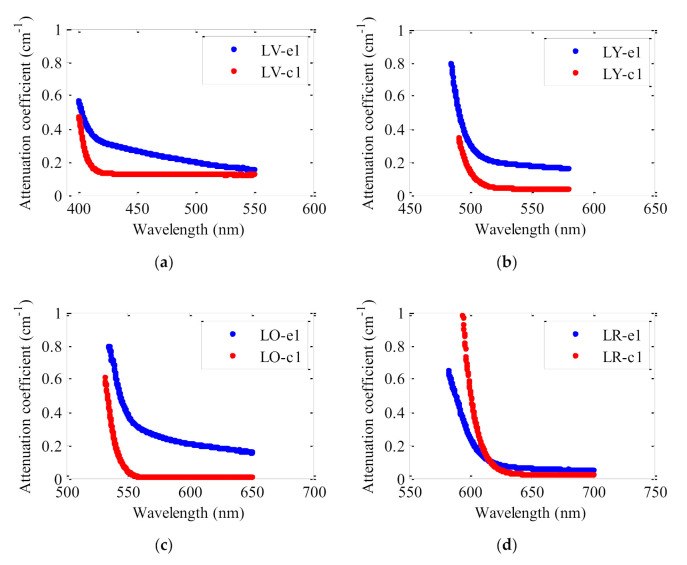
Attenuation coefficients for (**a**) LV, (**b**) LY, (**c**) LO and (**d**) LR doped fibers made by extrusion method (blue curves) and for those made by the casting one (red curves). All the fibers have the same dye concentration (0.003 wt%). The excitation irradiance was 22 nJ·cm^−2^ at 520 nm with *z*_e_ = 1.2 mm.

Figure 6 shows the attenuation coefficients obtained for the two types of fibers with the same dye concentration (0.003 wt%). These have been calculated by measuring the decrease of the irradiance of the fluorescence spectra as light propagation distance (*z*_ne_) increases with a constant excitation length. As can be seen the values of the attenuation curves of all the fibers made by the extrusion method are higher in the spectral regions where the dye absorptions tend to be negligible (regions with lowest slopes). Since the dopant concentration is the same in all fibers, the values of the attenuation coefficients at wavelengths outside the absorption band of the dyes most probably stem from the contribution of light scattering in the fibers. This suggests that light scattering is weaker in the fibers made by casting process. In fact, this method enables a more homogeneous dye distribution along the fibers compared to the extrusion method; since a fine controlled and closed chemical synthetic process, from monomer containing polymerization initiator, chain transfer agent and dopant to preform fabrication, guarantees a good dopant-monomer mixture solution, a non-bubble formation and the absence of impurities. In this method, all components are chemically mixed before polymerization occurs (preform formation), and the presence of chain transfer agent (1-butyl-mercaptan, BM) regulates the molecular weight of the polymer during the process. The use of mercaptans allows for fine control of the molecular chain length during polymerization, leading to easy processing conditions and mechanical performance of the resulting polymer. Then, imperfections that arise from microscopic variations in the material density induced by compositional fluctuations and by structural defects during fiber manufacturing processes are avoided. Moreover, the flat surfaces of borosilicate tubes force the material to have a rigid and polished surface along preform formation. On the contrary, in the extrusion method, a physical mixture of the components (PMMA and dopant), in the absence of solvent, is fed into an extruder, melted and then forced forward through the barrel by the rotating screw. This process makes the components slip slightly on the screw surface and keep on rotating while the screw continues to rub on the barrel surface. The molten mixture is then forced through a small nozzle, which shapes the resulting preform that solidifies during cooling. Consequently, a more randomly dopant distribution occurs during the continuous polymer-dopant feed process to the reactor, with a lower homogeneity of molecular weight distribution in the absence of chemical polymerization control. The preform fabrication by hand cranking also results in a rougher surface, and the viscous heating during the extrusion can induce the presence of microscopic defects, in the absence of well controlled heating-cooling process. Note that the desired extrusion temperature is rarely equal to the set temperature of the system. It is worth noting that this structural homogeneity and a flat preform’s surface-environment boundary are critical in the POFs fabrication where scattering due to the presence of imperfections induces optical losses by scattering.

**Figure 7:**
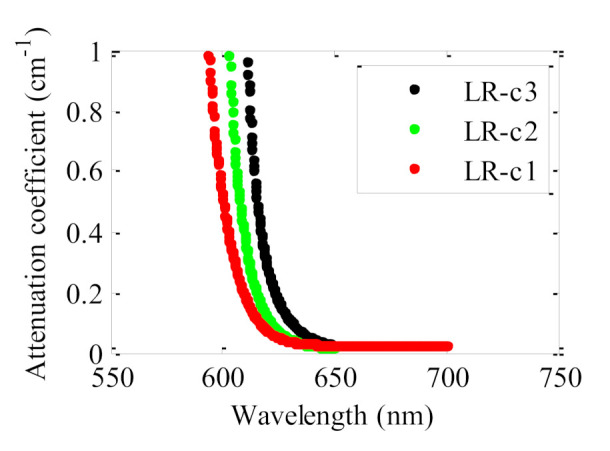
Attenuation coefficients for three lumogen red doped fibers manufactured using the casting method. The excitation irradiance was 22 nJ·cm^−2^ at 520 nm with *z*_e_ = 1.2 mm.

The attenuation coefficients of the fiber samples doped with LR with three different concentrations are plotted in Figure 7. As could be expected, the attenuation spectrum is shifted to longer wavelengths as the dopant concentration increases. This may be due to the creation of dopant aggregates as the dye concentration is increased [25]. We have not detected any dependence on the dye concentration of the attenuation coefficients corresponding to the spectral range where the dye absorption tends to zero.

It is difficult to compare the values displayed in Table 2 with other published values obtained in similar systems since they strongly depend on many factors, such as the physical parameters of the LSCs (dimensions, illuminated area,...), the experimental set-up used, the spectral range chosen to calculate of radiative powers or the employ of external devices [7,10,19,28,29,30,31]. In any case, the values indicate a poor performance of the fibers, with measured power values much smaller than the pump power ones. Besides the quality of the fibers, the low output signals of the analyzed fibers can be due to their low dye concentration, the short illuminated fiber-length (*z*_e_ = 5 cm), the absence of cladding in the fibers and the non-use of external devices. It is well-known that a thin cladding in a doped fiber causes a decrease in the transmission losses, and consequently, an increase in the overall efficiency [32]. Therefore, the output signal from the fibers could be easily improved by illuminating longer fiber-lengths, by employing higher dopant concentrations, by covering them with a thin cladding or by placing a mirror or a diffuser below them.

### 3.3. Photostability under Calibrated Solar-Simulator

**Figure 8:**
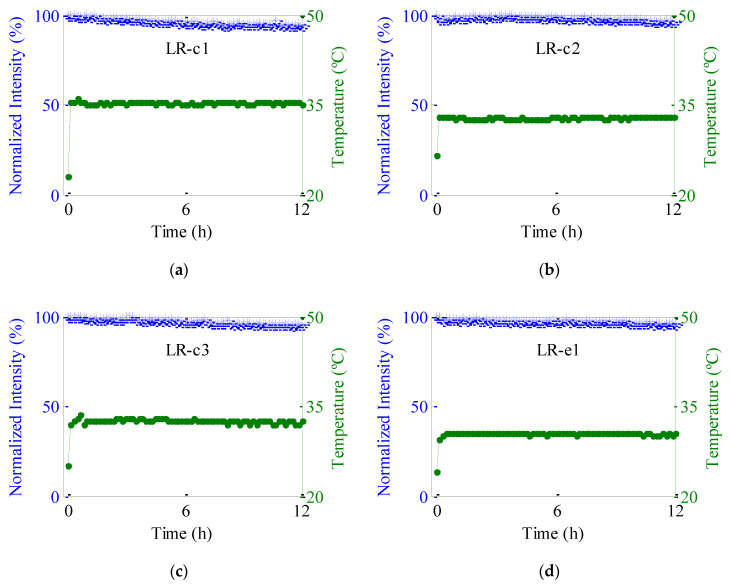
Normalized output intensity (blue crosses) and temperature (green circles) as a function of the sun-simulator exposition time for (**a**) LR-c1, (**b**) LR-c2, (**c**) LR-c3 and (**d**) LR-e1 samples. The green dashed lines of temperature data are guides to the eye.

The photostability of the total output power has been studied for the four fibers doped with lumogen red. The fiber samples were illuminated continuously for 12 h using a calibrated solar-simulator. In all the cases a fiber length of 5 cm was illuminated, except the sample with the highest LR concentration which was excited with *z*_e_ = 3 cm. For the sake of comparison, we have normalized the output power to 100% at the start of each measurement. The time evolution of the temperature of the fibers has also been measured. Figure 8 shows both results, that is, the obtained results for the normalized output power and the temperature.

It can be seen that the output signals from all samples are rather constant for this period of time, decreasing only around 5–7% from their initial values. This result agrees well with previous works that demonstrated that dyes derived from perylene exhibit long-term stability in PMMA hosts [23,33] and reinforces the high thermal stability of these lumogen-doped fibers. Moreover, these results may be considerably improved in real sunlight conditions since the light exposure has day/night cycles and it can undergo intensity variations. On the other hand, the temperature measurements indicate that the temperature of the fibers under the solar-simulator rapidly increases around 6–8 °C above the initial one. After that, the temperature stabilizes over the illumination time (around 35 °C). From the results obtained in the LR samples with the same concentration (LR c-1 y LR e-1), we can conclude that there is hardly any dependence of the photostability of the fibers on their manufacturing method. Similarly, the spectral analysis of the photostability of the fiber samples as a function of the exposure time does not depend on the manufacturing method. After 12 h of illumination, a very slight blue shift of the average wavelength of the emission spectra (around 0.4 nm) has been detected in all the samples analyzed, whereas the spectral width remains constant.

**Figure 9:**
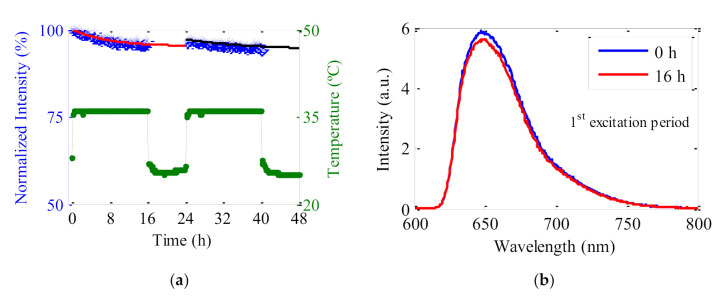
(**a**) Normalized output intensity (blue crosses) and temperature (green circles) of the LR-c3 fiber as a function of the sun-simulator exposition time. Red and black solid lines represent the fittings of Equation (2) to the intensity curves of both illumination periods. (**b**) Emission spectra of the sample at *t* = 0 and *t* = 16 h in the first excitation period.

We have also analyzed the effects of exposition on the LR-c3 fiber after a rest period of 8 h in darkness. The experiment was carried out for a total exposition time of 40 h, consisting in two light exposition periods of 16 h and a rest time of 8 h in between. The fiber was placed under the solar-simulator without changing its position along the whole experiment, so that the excited area remained invariable. The evolution of the normalized total output intensity and the emission spectra at the beginning and at the end of the first excitation period are shown in Figure 9.

During the first excitation period of 16 h the output signal of the fiber decreased slightly (about 5%) following an exponential decay. After maintaining the fiber in darkness for 8 h, it was illuminated again over a second period of 16 h. During this second period of radiation, the output intensity continued decreasing but at a lower rate (about 2% from its initial value). A very low signal recovery after the darkness period was detected (about 2%). The fittings of the two decays separately to an exponential expression of the following form:

(2) $$I(t)\;=a+\left[b\exp\left(-t/\tau \right)\right]\;$$

provides the values for *a*, *b* and *τ* constants shown in Table 3. In Equation (2), *a* constant indicates the normalized intensity at the end of the period, *b* provides the variation of the intensity in the period, and the value of *a* + *b* represents the intensity at the beginning of the period. *τ* is the decay time of the intensity emitted by the fiber. As can be seen from Table 3, the obtained values agree with the decays and the recovery estimated previously, and they confirm that photodegradation is lower in the second period. Although there is a significant error in the decay time τ of the second period, we can say that the fiber also undergoes a slow photodegradation in the second illumination time. 

## 4. Conclusions

In this work, the effect of the fabrication method of doped polymer optical fibers on their optical properties has been studied. Particularly, we have compared the performance as solar concentrators of four lumogen dye-doped polymer optical fibers self-fabricated using two types of fabrication processes: extrusion and casting. The study concludes that the lumogen dye-doped fibers fabricated by the casting process present better performance as solar luminescent concentrators than those fabricated by the extrusion process. This fact can, most probably, be due to a more homogeneous dye distribution and a more rigid and polished surface in the fibers made by the casting process, which cause lower optical losses in the light propagation through the fibers. Therefore, the fabrication process of the preform of the fibers is an important parameter in order to control the properties of POFs, in particular, their attenuation. On the other hand, the analysis of the photostability of fibers doped with different concentrations of lumogen red made by the casting method and one fiber doped with lumogen red fabricated by the extrusion one show slow decays of the output intensity of all the fibers, which reinforces the high thermal stability of these lumogen-doped fibers.

Although the performance of our self-fabricated lumogen dye-doped fibers working as solar luminescent concentrators is poor, it could be improved very easily by employing higher dopant concentrations or by placing reflecting surfaces below the fibers. There are other possibilities, such as the fabrication of fibers with a thin cladding, with other dye distributions, for instance hollow core fibers coated with doped material, or the use of a mixture of dyes as a dopant material. The idea of luminescent solar concentrators based on doped polymer optical fibers has emerged recently, and fortunately, there is still room for further improvement in their performance.

## Figures and Tables

**Table 1:** Properties of the fiber samples doped with lumogen dyes. e: extrusion method and c: casting method used to produce doped preforms. The fibers are step-index fibers and they have no cladding. *r* is the fiber radius.

Fiber Code	Type	Host	Dye	Concentration (wt%)	Diameter (2*r*) (mm)
LV-e1	SI Uncladded	PMMA	Lumogen F Violet 570	0.003	0.95 ± 0.03
LY-e1	SI Uncladded	PMMA	Lumogen F Yellow 083	0.003	0.81 ± 0.01
LO-e1	SI Uncladded	PMMA	Lumogen F Orange 240	0.003	1.00 ± 0.03
LR-e1	SI Uncladded	PMMA	Lumogen F Red 305	0.003	0.95 ± 0.05
LV-c1	SI Uncladded	PMMA	Lumogen F Violet 570	0.003	0.74 ± 0.01
LY-c1	SI Uncladded	PMMA	Lumogen F Yellow 083	0.003	0.87 ± 0.01
LO-c1	SI Uncladded	PMMA	Lumogen F Orange 240	0.003	0.99 ± 0.01
LR-c1	SI Uncladded	PMMA	Lumogen F Red 305	0.003	0.91 ± 0.01
LR-c2	SI Uncladded	PMMA	Lumogen F Red 305	0.01	1.01 ± 0.01
LR-c3	SI Uncladded	PMMA	Lumogen F Red 305	0.03	0.93 ± 0.01

**Table 2:** Output intensities and powers, optical efficiencies (%) and concentration factors *C* of the fibers doped with lumogen dyes calculated with an illuminated fiber length *z*_e_ = 5 cm.

Fiber Code	*I*_out_ (μW/cm^2^)	*P*_out_ (μW)	*η*_opt_ (%)	*C*
LV-e1	22.72	0.16	3.39 × 10^−4^	2.27 × 10^−4^
LV-c1	44.07	0.19	5.12 × 10^−4^	4.41 × 10^−4^
LY-e1	51.66	0.27	6.57 × 10^−4^	5.17 × 10^−4^
LY-c1	79.80	0.47	1.09 × 10^−3^	7.98 × 10^−4^
LO-e1	61.37	0.48	9.64 × 10^−4^	6.14 × 10^−4^
LO-c1	80.97	0.62	1.26 × 10^−3^	8.10 × 10^−4^
LR-e1	50.34	0.36	7.51 × 10^−4^	5.03 × 10^−4^
LR-c1	92.64	0.72	1.46 × 10^−3^	9.26 × 10^−4^
LR-c2	196.55	1.33	2.87 × 10^−3^	1.97 × 10^−3^
LR-c3	397.23	3.12	6.24 × 10^−3^	3.97 × 10^−3^

**Table 3:** Values obtained from the fittings to the first and to the second period of the experimental curve of Figure 9 with Equation (2). *R*^2^ is the coefficient of determination of the fitting.

	*a*	*b*	*a + b*	*τ* (min)	*R* ^2^
1st period	95.2 ± 0.3	4.9 ± 0.3	100.1 ± 0.6	450 ± 60	0.89
2nd period	94 ± 1	3.1 ± 0.9	97 ± 2	900 ± 400	0.68

## Data Availability

Not applicable.
